# Cardiofaciocutaneous Syndrome Type 4 due to a MAP2K2 Variant: Expanding the Phenotypic Spectrum With Feeding Dysfunction and Neurodevelopmental Involvement

**DOI:** 10.1002/ccr3.72024

**Published:** 2026-02-11

**Authors:** Aleksandra Świeca, Małgorzata Rydzanicz, Rafal Ploski, Krzysztof Szczałuba

**Affiliations:** ^1^ Department of Medical Genetics, Medical University of Warsaw 02‐106 Warsaw, Poland; Center of Excellence for Rare and Undiagnosed Disorders Medical University of Warsaw Warsaw Poland; ^2^ Department of Medical Genetics Medical University of Warsaw Warsaw Poland

**Keywords:** Cardiofaciocutaneous syndrome type 4, CFC4, MAP2K2, neurodevelopment, RASopathy

## Abstract

We report a female infant with cardiofaciocutaneous syndrome type 4 (CFC4), an ultra‐rare RASopathy caused by a heterozygous *MAP2K2* (c.619G>A, p.Glu207Lys) variant. From birth, she presented with neonatal hypotonia, respiratory distress, and feeding dysfunction characterized by absent sucking reflex, orofacial hypotonia, and sensory disturbances. Distinct dysmorphic features, including characteristic craniofacial anomalies and macroglossia, were noted. Cardiac evaluation revealed a patent foramen ovale and two small atrial septal defects. Neurologic manifestations included cyanotic apnea, dystonic stiffening, and tremor. Electroencephalography demonstrated bilateral temporoparietal epileptiform discharges, and brain MRI revealed reduced cerebral white matter volume. Although epilepsy has not been definitively diagnosed, the patient remains under ongoing neurological surveillance. Cutaneous involvement included xerotic, papular skin with multiple pigmented nevi and segmental hemangiomas. Ophthalmologic evaluation demonstrated hyperopia with astigmatism. Metabolic assessment suggested mild energetic dysfunction, with elevated triglycerides and citric acid cycle intermediates, without evidence of a defined inborn error of metabolism. Over time, the patient developed persistent feeding difficulties with choking due to oropharyngeal dysfunction, sleep disturbances, delays in gross and fine motor development, and behavioral dysregulation, including aggressive and self‐injurious behaviors. Despite the preserved social interest and frequent social approach toward unfamiliar individuals, pronounced anxiety and distress were observed during caregiver absence or reduced attention. This case expands the clinical spectrum of CFC4 associated with MAP2K2 variants, highlighting early feeding dysfunction, paroxysmal neurologic events, and prominent sleep and behavioral disturbances as key diagnostic features.

## Key Clinical Message

This case extends the understanding of MAP2K2‐related cardiofaciocutaneous syndrome type 4 (CFC4) beyond its hallmark craniofacial, cutaneous, and cardiac features. It highlights feeding dysfunction, sleep abnormalities, and developmental delay as equally significant components of the phenotype, underscoring the need for comprehensive, multidisciplinary management in CFC.

## 1 Introduction

Cardiofaciocutaneous (CFC) syndrome is a rare RASopathy resulting from pathogenic variants in genes that regulate the RAS/MAPK pathway, which is essential for cellular growth and differentiation. Most cases are associated with *BRAF* variants, but variants in *MAP2K1*, *KRAS*, and *MAP2K2* have also been identified [1]. As this intracellular cascade plays a central role in controlling cell growth, differentiation, and survival, its dysregulation leads to various developmental abnormalities. CFC type 4 (OMIM 615280), caused by *MAP2K2* variants, represents one of the rarest molecular subtypes, with limited phenotype‐specific data. Characteristic features of CFC include macrocephaly, high forehead, curly or sparse hair, hyperkeratotic skin, segmental hemangiomas, and cardiac defects such as pulmonary stenosis or septal anomalies [2, 3]. Neurological involvement, especially hypotonia, seizures, and global developmental delay, is frequent [1]. Feeding difficulties in infancy are often severe and may be the earliest clinical manifestation [4]. Here, we report an infant with a heterozygous *MAP2K2* variant (c.619G>A, p.Glu207Lys), presenting from birth with oromotor dysfunction, macrosomia, distinctive craniofacial and cutaneous features, and paroxysmal neurologic episodes, thereby expanding the early phenotypic spectrum of CFC type 4.

## 2 Case History

The patient is a female infant born from the third pregnancy via elective cesarean section at 38 weeks and 5 days. Antenatal history included gestational hypertension; however, prenatal screening and ultrasounds were unremarkable, with a low trisomy risk. Delivery was complicated by thick meconium‐stained fluid and a nuchal cord. The neonate was hypotonic, had poor spontaneous activity, profuse secretions, and Apgar scores of 6 both at 1 and 5 min. Birth weight was 4300 g (+2.65 SD), head circumference 37 cm (+2.91 SD). Postnatally, she developed respiratory distress requiring NICU admission and six days of nasal CPAP. Clinical examination revealed profound hypotonia, weak primitive reflexes, peripheral cyanosis, and tachypnea up to 90/min. Chest radiography showed bilateral perihilar and pericardiac infiltrates. Cranial ultrasound on day 3 revealed grade I intraventricular hemorrhage; abdominal ultrasound was normal. Infectious workup (TORCH screen, CMV, blood cultures) and inflammatory markers (CRP, PCT, IL‐6) were unremarkable. A complete blood count revealed thrombocytosis (301 × 10^3/μL), erythroblasts (3/100 leukocytes), anisocytosis, microcytosis, and polychromatophilia. A systolic murmur was detected on day 3; echocardiography revealed a patent foramen ovale (PFO) with left‐to‐right shunting and two minor atrial septal defects near the superior vena cava. She is the third child of non‐consanguineous parents. Maternal history includes psoriasis and obesity (BMI 34.05); the father has multiple sclerosis. The older brother has autism spectrum disorder and the sister has a diagnosed PFO. No family history of neurocutaneous syndromes or developmental disorders was reported.

## 3 Differential Diagnosis, Investigations, and Treatment

**FIGURE 1:**
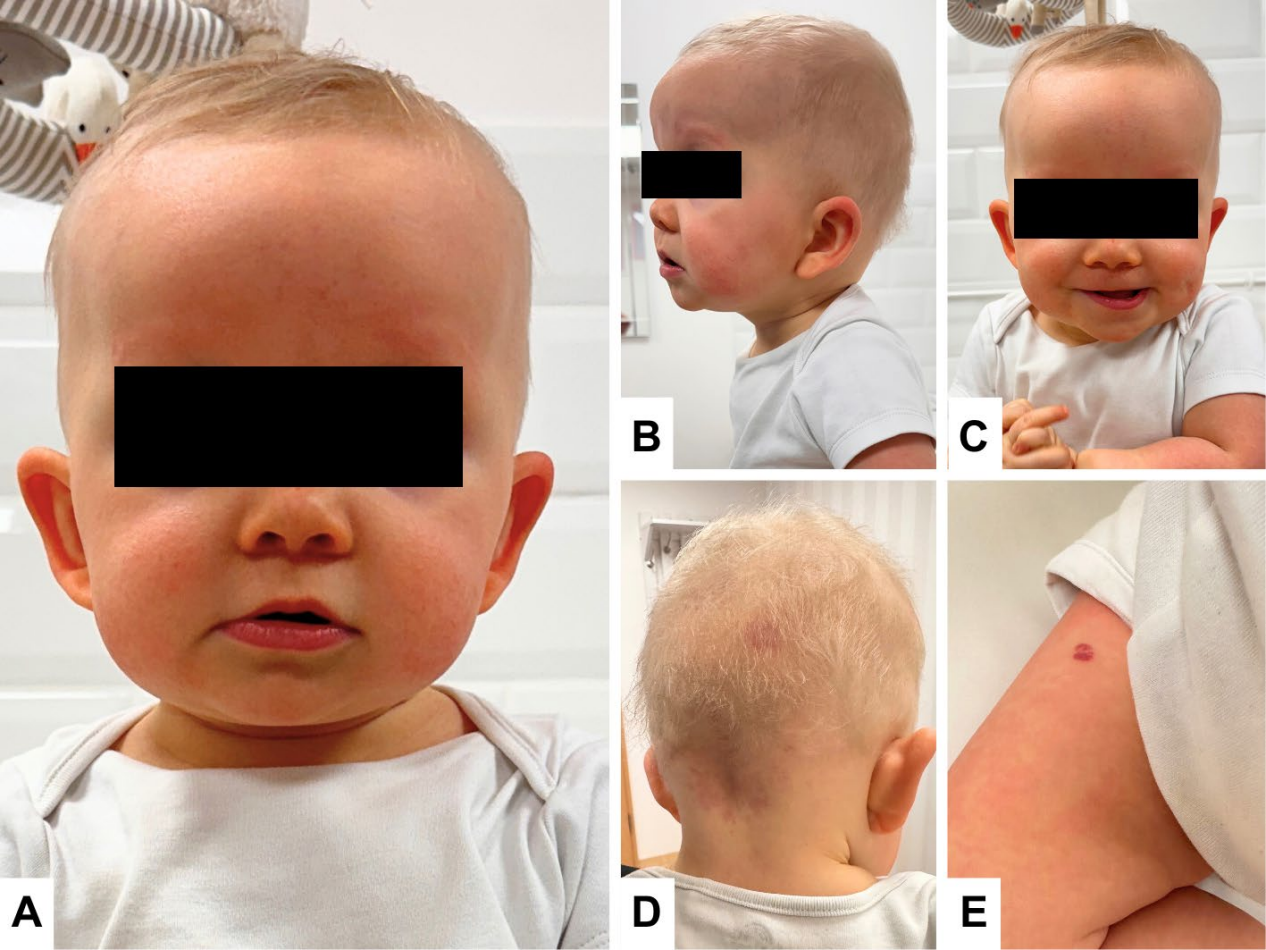
Dysmorphic features and cutaneous findings in a 13‐month‐old girl with CFC4. (A–C) Facial dysmorphism, including a prominent, high forehead; depressed nasal bridge; bulbous nasal tip; epicanthus; underdeveloped supraorbital ridges; low‐set and posteriorly rotated ears; and marked absence of eyebrows and eyelashes. (D) Segmental hemangioma in the occipital region. (E) Segmental hemangioma on the right thigh.

Physical examination during the genetic consultation revealed macroglossia, segmental hemangiomas (present on the forehead, scalp, thigh, and labia), macrosomia, and facial dysmorphism, including a round face, a depressed nasal bridge, a broad nasal tip, epicanthal folds, and prominent earlobes with posterior creases (Figure 1). Neurologic evaluation showed generalized hypotonia with symmetric deep tendon reflexes. Feeding difficulties (choking, gagging, ineffective suck) were present from birth. Due to the phenotype, Beckwith–Wiedemann syndrome (BWS) was initially suspected. Genetic testing for BWS was negative. Whole‐exome sequencing performed at 5 months revealed a heterozygous c.619G>A (p.Glu207Lys) variant in the *MAP2K2* (*MEK2*) gene, confirming autosomal dominant CFC4.

At 6 months, the oral motor assessment revealed an absent sucking reflex, preserved cough, macroglossia with vertical tongue movement, facial hypersensitivity, intraoral hyposensitivity, and hypotonia of the lips, cheeks, and neck. Feeding therapy and neuromotor rehabilitation were initiated. At 8 months, the weight was 8.47 kg (−0.2 SD) and head circumference was 47 cm (+2.6 SD). She remained hypotonic, unable to sit, crawl, or roll independently. In the prone position, she achieved high support but fatigued quickly, likely due to macrocephaly.

Her history included a single cyanotic episode with apnea lasting approximately one minute, described by parents as wide‐eyed, open‐mouthed, with deep purplish‐red discoloration, mimicking choking. No aspiration or foreign body was found. At 4 months, she had one episode of whole‐body tremor (“like a vibrating phone”) with preserved consciousness and no postictal signs. At 11 months, she developed daily clusters of transient stiffening with skin discoloration lasting approximately 3 s and nightly episodes of inconsolable crying with vomiting. Electroencephalography at 10 months demonstrated bilateral temporoparietal sharp–slow wave complexes. Brain MRI at 12 months revealed reduced cerebral white matter volume.

Ophthalmologic examination showed mild‐to‐moderate hyperopia with astigmatism (approximately +1.75 to +2.0 diopters), for which corrective lenses were prescribed at 15 months of age. Fundoscopy revealed normal optic discs and retinal vasculature without evidence of progressive pathology, and retinal attachment was confirmed on ocular ultrasound. Intermittent convergent strabismus of the left eye was observed, predominantly during fatigue.

At 16 months of age, metabolic evaluation revealed elevated triglyceride levels and increased 2‐ketoglutaric acid, along with elevation of other citric acid cycle metabolites, interpreted as suggestive of mild energetic dysfunction, without evidence of a defined inborn error of metabolism.

Her skin was dry and papular, requiring twice‐daily emollient therapy. Multiple small pigmented nevi (approximately 10–15) were noted, with the gradual appearance of new lesions over time. Previously identified hemangiomas persisted; the largest occipital lesion showed mild fading, and no new lesions appeared. Marked macroglossia with resting tongue protrusion was observed. Dental development was otherwise age‐appropriate, although an anterior crossbite was present at 19 months, and bruxism occurred during sleep and periods of emotional distress.

At 21 months of age, feeding difficulties persisted despite good appetite and tolerance of a wide range of foods. She continued to be breastfed and most often fell asleep during breastfeeding. Refusal was frequently followed by episodes of intense, inconsolable crying and panic. Oropharyngeal dysfunction was evident, manifested by persistent drooling, daily choking episodes, and occasional severe events requiring caregiver intervention. Although she was able to bite and chew, she frequently attempted to swallow unchewed food. Choking also occurred during liquid intake, particularly when drinking independently. Growth parameters at that time showed a weight of 11 kg (+0.03 SD), length of 78 cm (−1.99 SD), and head circumference of 51 cm (+3.01 SD), indicating disproportionate growth with macrocephaly and reduced linear growth.

Motor evaluation demonstrated delayed gross motor development. Independent sitting was achieved at 16 months, crawling at 17 months, and cruising at furniture at 18 months; a few unsupported steps were possible, although gait remained unsteady. Truncal and gait ataxia, frequent falls, rapid fatigability, and episodes of loss of balance were observed. Generalized hypotonia persisted, accompanied by ligamentous laxity and joint hypermobility. Fine motor skills were also delayed. Play was predominantly nonconstructive, without purposeful stacking or manipulation of objects. She was able to clap and wave and showed interest in drawing; however, she had difficulty maintaining a stable grasp and applying sufficient pressure.

Sleep was assessed using the revised Brief Infant Sleep Questionnaire (BISQ‐R), yielding a total score of 33.16 at 19 months of age [5]. Clinically, sleep was characterized by significant disturbances, including restless sleep and frequent nocturnal awakenings (approximately four per night). Parents reported recurrent brief nocturnal apneic episodes lasting 10–15 s, followed by gasping respirations and crying. Episodes consistent with night terrors were observed, characterized by apparent fear, generalized stiffening, and flexion of the lower limbs. She did not nap during the day and had a markedly prolonged sleep onset, typically requiring approximately three hours to fall asleep after the lights were turned off.

At 21 months, the Brief Infant–Toddler Social and Emotional Assessment (BITSEA) showed a Competence score of 14, meeting criteria for delayed competence, and a Problem score of 32, exceeding the threshold for clinically significant behavioral concerns [6]. Stereotypic movements were present, including spinning in circles and hand flapping. Additionally, she exhibited daily episodes of aggressive and self‐injurious behaviors. Self‐directed behaviors included hair pulling, scratching, biting of hands, and hitting herself on the head. These behaviors appeared abruptly, persisted over time, and were particularly pronounced during states of sensory overload or emotional overstimulation.

Despite these difficulties, social engagement was preserved. She was socially open, frequently smiled, and readily approached and interacted with unfamiliar individuals, with a strong desire for social engagement. Expressive language consisted of approximately eight spoken words at 21 months of age. Although socially open and engaging, the patient showed pronounced anxiety when caregivers were not present and in situations where attention was withdrawn, leading to significant distress and inconsolable crying.

## 4 Conclusion and Results

The patient is currently under multidisciplinary care, including cardiology follow‐up for surveillance of hypertrophic cardiomyopathy, ophthalmologic care, surgical monitoring of hemangiomas, and gastroenterology evaluation due to recurrent choking episodes. She also participates in intensive rehabilitation and therapeutic interventions, attending structured therapy sessions five times per week. Current management includes supplementation with vitamin D, coenzyme Q10, and omega‐3 fatty acids.

The patient's main clinical challenges include global neurodevelopmental delay, with prominent impairments in gross and fine motor skills, speech development, and behavioral regulation. Daily functioning is substantially affected by sleep disturbances, aggressive and self‐injurious behaviors, and persistent feeding difficulties due to oropharyngeal dysfunction with frequent choking.

## 5 Discussion

CFC4 is a rare RASopathy caused by pathogenic variants in *MAP2K2* [1]. Although the broader clinical spectrum of CFC is well‐characterized, the phenotype associated with *MAP2K2* remains incompletely defined due to limited case numbers. The c.619G>A (p.Glu207Lys) variant detected in our patient is absent from population databases and has been reported in at least six individuals with RASopathy features, supporting its pathogenicity.

Our report expands the phenotypic spectrum of CFC4 by documenting severe early feeding dysfunction, macrosomia, and paroxysmal neurologic episodes together with hallmark craniofacial, cutaneous, and cardiac manifestations. While epilepsy was not confirmed, dystonic stiffening and whole‐body tremors highlight the need for ongoing neurological surveillance. Seizures occur in up to 64% of CFC patients, usually as generalized tonic–clonic or absence types, with fewer reports of complex partial seizures or spasms [7]. In general, *MAP2K2* variants are associated with milder seizures and better outcomes than *BRAF* or *MAP2K1* mutations [8]. Neuroimaging abnormalities, as in our patient with reduced cerebral white matter, are frequent but nonspecific, with ventriculomegaly or hydrocephalus occurring in up to two‐thirds of cases [9].

Furthermore, feeding difficulties are a common and frequently persistent feature of CFC, significantly impacting the quality of life. Orofacial hypotonia and hypersensitivity contribute to oral aversion, gagging, and an increased risk of aspiration [4]. These difficulties may necessitate enteral feeding or targeted therapeutic interventions.

Cardiac involvement is a hallmark of CFC syndrome, reported in 71% of cases [10]. The most common cardiac manifestations include pulmonary valve stenosis, hypertrophic cardiomyopathy (HCM), atrial septal defects, and ventricular septal defects [10]. HCM is currently managed primarily with propranolol, which remains the first‐line therapy in affected children [11]. Ongoing clinical studies are evaluating the potential role of trametinib, a MEK inhibitor, as a targeted treatment for CFC‐related HCM (NCT06555237).

As no targeted treatments are currently available, multidisciplinary care across neurological, cardiac, dermatological, gastrointestinal, and developmental domains remains essential for effective management [1]. Emerging data suggest that repurposing MEK inhibitors (e.g., trametinib, selumetinib), currently approved in oncology, may offer a potential treatment option for RASopathies [12]. As we may be approaching a breakthrough in targeted management of this group of disorders, continuous documentation of clinical presentations remains crucial for refining the phenotypic spectrum of CFC4 and guiding future therapeutic strategies.

## Author Contributions

**Aleksandra Świeca:** investigation, writing – original draft. **Małgorzata Rydzanicz:** data curation, investigation. **Rafal Ploski:** supervision. **Krzysztof Szczałuba:** conceptualization, supervision, validation, writing – review and editing.

## Funding

The authors have nothing to report.

## Ethics Statement

This study was approved by the Bioethical Committee of the Medical University of Warsaw (Approval Code: KB/142/2021, issued on October 4, 2021).

## Consent

Written informed consent for publication of the clinical details and accompanying photographs was obtained from the patient's legal guardian.

## Conflicts of Interest

The authors declare no conflicts of interest.

## Data Availability

Data sharing not applicable to this article as no datasets were generated or analysed during the current study.
